# Modern Sociology

**Published:** 1899-05-06

**Authors:** 


### Modern Sociology

### PITY THE POOR LANDLORD.

One of the favourite outcries of those papers which draw on the sympathies of the rich by dilating on the sorrows of the poor, is that slum property is rackrented. Sometimes this statement is true enough; but, for the sake of fairness, it is well to point out that the fact that a block of workmen's tenements brings in more gross rental than a villa or mansion covering the same area does not prove that the working man is rack-rented. A house cannot be rated nor rented solely in proportion to the ground it covers. There is, in the first place, the respective values of sites. Without going into the delicate question of who ought to receive the increased value of ground that has become more precious through no act of the owners, the fact remains that certain pieces of land are worth more than others, and, whether paid to a landlord or to the State, ground rent must always be reckoned upon.

Apart from this, however, there are other expenses connected with the housing of the poor. A block occupied by a score of families, instead of four or five, must have a corresponding increase in kitchen ranges, laundries, sanitary conveniences, and so forth. And it is just these parts of a house that are most apt to get out of order, as every householder knows. Add to this that the tenants, though sometimes house-proud after their fashion, use their dwellings more roughly than more refined people do theirs. Even in the proverbial "best-regulated households" the nursery needs to be done up more frequently than the drawing-room. In the house of the working-man you may safely assume that every room is a nursery; likewise the stairs and courts, and, in short, every bit of the building. Thence come holes in the plaster, bruises and splinters in the woodwork, and the continual defilement and destruction of all paint and varnish. To keep these things up to even a modest standard of decency implies a very considerable expense. If they are neglected the property soon gets very dilapidated, and the repairs are proportionately more costly. A bad landlord, who is a foolish one also, grudges these repairs, with the result that his best tenants leave him, and his worst, who pay him the compliment of remaining on, try to pay him little else. Rack-rented they may be, but as they don't pay their rents this troubles them little. It must, too, always be remembered that in many cases where high rents are asked for only a fraction of these can be actually collected. And, of course, it is the family whose means and intention of paying are most wanting that are most willing to promise any rent that may be asked.

Still, these repairs and the expense implied in making them and keeping the place in satisfactory condition are within the landlord's option to yield. But repairs connected with sanitation must be attended to, whether the landlord like it or not, and it is these repairs which are the most constant and expensive. Householders among the poor seem to have the most excessive notions of what a little drain can do. It is "out of sight out of mind" with them, and they feel deeply aggrieved when the drain down which was thrust a dress-bodice or a pair of old shoes yesterday is flooded and will not work to-day. When the water supply is good, a well-made drain will indeed carry away a wonderful amount of solid matter of all sorts. But the poor, as a rule, have very little idea of the importance of a good flow of water, and when once the offensive matter or the rags and scraps discarded from the household disappear from sight, they think that all is well. Thence arise troubles innumerable, and these troubles the landlord must cure. If a sanitary inspector notes that any part of the premises is in an unhealthy condition, a notice is served on the landlord that a nuisance exists in his property, and he is given a short time—as a rule, a very short time—in which to have it removed. In some places it would almost be necessary to keep a plumber on the premises; in every one it will be found that a tenement is better kept when there is an intelligent man, possessed of judgment and some skill with his hands, employed as a resident caretaker.

Add to this the difficulty that is found in collecting rents. The middle-class tenant has, as a rule, the money waiting for the landlord when he calls, the richer often send a cheque in advance; but among the poor the visit of the landlord or his representative when the rent is due often means long interviews, explanations, excuses, promises, but very little money in hand. Much harrying and worrying, threatening and summoning, are required to obtain the money, even if the tenant does not make a "moonlight flitting" while negotiations are in progress. The rents in the dwellings of the poor have to be collected more frequently than in better-class houses, hence there is more trouble for the landlord, or, if he employs a substitute, its equivalent in expense. You can get a man to collect the rents of a respectable property for a much smaller commission than he would demand for one of poorer class.

Now all this the landlord takes account of, and must take account of, in fixing the rent, if his property is to be a profit to him, and not a loss as well as a continual trouble. When all deductions are made, it will be found that the profit is not so great after all, not by any means so great as one who simply compared the original cost of the building with the gross rental might think. Of course, there are good landlords and bad; some who do the best they can for their tenants, others who try only to get as much out of them as they can. The average landlord does not spend his life trying to grind the faces of the poor, in spite of all that is said. But there is one charity that a wise landlord, however good and kind-hearted he may be, will never indulge in. He never remits a rent. However reasonable the excuse may be for the money not being forthcoming in one particular case, he knows that if in it he forbears to exact his pound of flesh, on his next visit he will find that at least half of his tenants have a pitiful tale to tell of children ill, of husbands out of work, or something else that justifies non-payment. And if he refuses to let these tenants live rent-free they will all reproach him with his previous kindness to their neighbour. He may, if he is convinced that the case is necessitous and deserving, cause some help to be given; but he takes care that his name does not appear in it, and he never, never remits a rent. If he is not running his property as a charity, and a charity of which not all the recipients are worthy, he must give his donations with the strictest regard for the Scriptural injunction not to let his right hand know the doings of his left. Therefore, let those who condemn the landlord of a poor man's house because the rents he asks seem to be high, consider these things before coming to the conclusion that he is a heartless and conscienceless oppressor of the poor.

